# Survival prediction of colorectal cancer using 101 machine learning methods based on immune-related genes: A machine learning study

**DOI:** 10.1097/MD.0000000000048757

**Published:** 2026-05-22

**Authors:** Binyan Liu, Huimin Li, Mengdi Hao, Xiaoli Liu, Dajin Yuan, Wenbin Huang, Wenjie Li, Jia Zeng, Yubing Zhu, Lei Ding

**Affiliations:** a Department of Gastrointestinal Surgery, Beijing Shijitan Hospital, Capital Medical University, Beijing, China.

**Keywords:** colorectal cancer, immune-related genes, IRGRS, machine learning model, survival prediction

## Abstract

Colorectal cancer (CRC) is characterized by high incidence and mortality rates. Early identification of high-risk CRC patients and timely intervention are crucial for reducing mortality rates. This study aims to develop a prognostic model incorporating immune-related genes using 101 machine learning algorithms. We utilized data from 630 CRC patients in The Cancer Genome Atlas Program database and the Gene Expression Omnibus database as a validation set. We identified immune-related genes from the ImmPort repository and conducted differential gene expression analysis. We developed 101 machine learning models, including random survival forest, elastic net, LASSO, Ridge regression, stepwise Cox, Coxboost, partial least squares regression for Cox, supervised principal component analysis, generalized boosted modeling, and survival support vector machines. The model with the highest mean concordance index was selected as the optimal prognostic model. The Ridge regression model demonstrated the most robust performance with a mean concordance index of 0.67. Patients with higher immune-related gene risk score (IRGRS) had significantly lower survival rates (log-rank test, *P* < .001). Univariate and multivariate Cox regression analyses confirmed IRGRS as an independent prognostic factor. Nomograms successfully predicted overall survival at 1, 2, and 3 years. IRGRS correlated with immune cell infiltration levels and stromal activity in the tumor microenvironment. High IRGRS scores were associated with higher tumor immune dysfunction and exclusion scores, suggesting potential differences in response to immune checkpoint inhibitors. In addition, high IRGRS patients exhibited greater sensitivity to certain chemotherapy drugs. The IRGRS score is a potent predictor of survival in CRC patients. This study offers a new perspective for CRC prognosis and supports the development of personalized treatment strategies.

## 1. Introduction

According to statistics from the International Agency for Research on Cancer, there were over 1.9 million new cases of colorectal cancer (CRC) globally in 2020, with over 930,000 deaths.^[1]^ The high mortality rate of CRC is largely due to the rapid progression of the disease and inadequate treatment. Therefore, early identification of high-risk CRC patients and timely intervention and treatment are crucial for reducing mortality rates.^[2]^

Despite existing research that has focused on the expression of individual genes to explore the impact of specific gene mutations on the prognosis of CRC, significant results have been achieved, such as the milestone discoveries of mutations in the *P53* gene and the *K-ras* gene.^[3,4]^ However, investigators are increasingly recognizing that CRC is a highly complex disease, and the ideal biomarker should have homogeneous expression within and between tumor tissues to ensure stable performance in all patients.^[5]^ Therefore, multigene combinations may be a promising approach to address this issue of heterogeneity. With the advancement of bioinformatics technology, many prognostic gene signatures have been developed to identify the risk level of patients’ diseases and correlate with prognosis.^[6,7]^ However, due to improper machine learning methods, a lack of rigorous validation across different cohorts, and the absence of clinical testing, multigene expression signatures often face challenges in clinical application.

In recent years, advancements in immunotherapy research have provided new insights for the treatment of CRC.^[8,9]^ Immune-related genes (IRGs) play a crucial role in the human immune system, participating in the regulation of immune responses, including the recognition and elimination of pathogens, tumor cells, and other foreign substances, as well as maintaining immune tolerance to prevent the occurrence of autoimmune diseases.^[10,11]^ Due to the heterogeneity of tumors, there is a significant variability in immunotherapy responses among individuals. Therefore, there is an urgent need to identify the tumor microenvironment (TME) and immune infiltration characteristics of CRC and to develop predictive models for patient treatment response and prognosis.

In this study, we aim to construct 101 machine learning models, utilizing data from 630 CRC patients in The Cancer Genome Atlas Program (TCGA) database, to develop and validate a risk stratification label. In addition, we will use data from the Gene Expression Omnibus (GEO) database as a validation set to assess the potential of the immune-related gene risk score (IRGRS) in predicting the prognosis of CRC. We will also investigate the correlation between the IRGRS score and immune cell infiltration, as well as the TME. This may help to optimize precision treatment strategies and further improve the clinical outcomes of patients with CRC.

## 2. Materials and methods

### 2.1. Data acquisition

We procured clinical data from 630 CRC patients enrolled in the TCGA project, specifically from the TCGA-READ and TCGA-COAD cohorts. The dataset encompassed a comprehensive range of parameters, including messenger ribonucleic acid expression profiles, survival durations, statuses, demographic details, pathological staging according to tumor, node, metastasis classification, and genomic mutation profiles. In addition, we accessed RNA sequencing data from the GEO database (http://www.ncbi.nlm.nih.gov/geo/), GSE39582 series, which comprises 585 CRC samples along with their corresponding detailed clinical annotations. To quantify the tumor mutational burden, we leveraged mutational data extracted from TCGA. All data used for this study were obtained from a public database; therefore, the requirements for ethical approval and patient consent were waived.

### 2.2. Acquisition of IRGs

The inventory of IRGs was sourced from the ImmPort repository (https://immport.org/). Employing the limma R package, we conducted a differential gene expression analysis to pinpoint genes exhibiting statistically significant expression disparities between tumor and normal tissues. The selection criteria were stringent: log-fold change >1 and a *P*-value threshold of <.05. Subsequently, a heatmap was constructed to visually represent these differentially expressed genes. To further refine our gene list, we conducted survival analyses on the shortlisted genes, thereby identifying those with prognostic significance.

### 2.3. Machine learning

We harnessed a panel of 10 machine learning algorithms to integrate the genes previously identified, encompassing random survival forest, elastic net, LASSO, Ridge regression, stepwise Cox, Coxboost, partial least squares regression for Cox, supervised principal component analysis, generalized boosted modeling, and survival support vector machines. A consensus model was derived from these methodologies. Employing a leave-one-out cross-validation strategy, we evaluated 101 distinct algorithmic combinations to refine our prognostic models. The TCGA dataset served as our training cohort, with the GSE39582 dataset utilized for validation. An IRGRS was calculated for each cohort, based on the predictive signatures from the training dataset. The model exhibiting the highest mean concordance index (C-index) was adjudged as the optimal prognostic model.

### 2.4. Prognostic dissection of high and low IRGRS cohorts

The TCGA cohort was stratified into high and low IRGRS score groups using the median score as the discriminating threshold. Survival analysis was performed using Kaplan–Meier estimators, visualized through the survminer and survival R packages. Both univariate and multivariate Cox regression analyses were implemented to assess the prognostic significance of the IRGRS. To enhance the precision of overall survival predictions, we integrated clinical covariates with the IRGRS within a nomogram framework, analyzed and visualized using the rms package. In addition, calibration plots were generated to appraise the congruence between predicted and observed survival outcomes. The predictive accuracy of our model was quantified using the C-index and the area under the curve.

### 2.5. Immune infiltration analysis

We utilized a suite of algorithms, including CIBERSORT-ABS, CIBERSORT, EPIC, MCPCOUNTER, QUANTISEQ, XCELL, and TIMER, to quantify the heterogeneity in immune cell infiltration between cohorts stratified by high- and low-risk scores. The corrplot package facilitated the exploration of correlations between the risk score and immune cell populations. Employing the ESTIMATE algorithm, we determined the abundance of immune cells (immune score) and stromal cells (stromal score). To further delineate differential immune cell infiltration patterns and immunological functions, we applied the GSEABase package and conducted single-sample gene set enrichment analysis. In addition, we leveraged the tumor immune dysfunction and exclusion (TIDE) score, available at http://tide.dfci.harvard.edu, to forecast the potential responsiveness to immunotherapeutic interventions.

### 2.6. Gene set enrichment analysis

Using the clusterProfiler R package, gene set enrichment analysis (GSEA) was conducted with “c2.cp.kegg.Hs.symbols.gmt” and “c5.go.Hs.symbols.gmt” as reference gene sets to analyze the diversity of signaling pathways between patients with high and low IRGRS scores. The limma package was utilized to calculate the log-fold change values for genes. The top 5 enriched gene sets were selected and presented.

### 2.7. Analysis of immune checkpoint and chemotherapy drug sensitivity

A comparative analysis of immune checkpoint molecule levels across different patient groups was performed. The Genomics of Drug Sensitivity in Cancer 2 methodology was harnessed to assess patient responses to chemotherapy. Furthermore, the oncoPredict package was employed to evaluate chemotherapeutic sensitivity based on the 50% maximum inhibitory concentration, providing insights into drug efficacy.

## 3. Results

### 3.1. Identification of crucial immune-related genes

**Figure 1.**
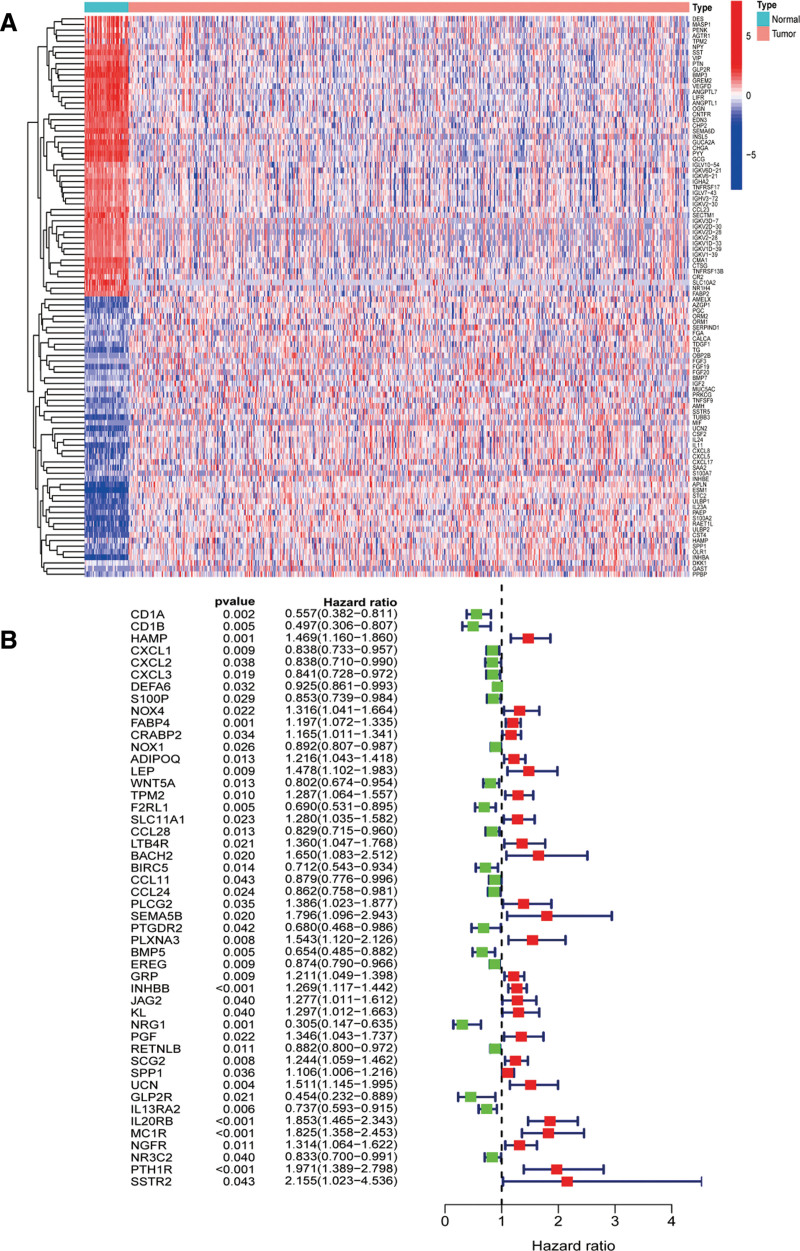
(A) Heat map of differentially expressed genes. (B) Forest plots of hazard ratios derived from 48 prognostic related genes.

We procured a comprehensive list of IRGs from the ImmPort database. Subsequently, employing the limma R package, we identified 502 differentially expressed genes, which were visually represented as a heatmap (Fig. 1A). Through survival analysis, we further narrowed down the list to 48 genes with significant prognostic value (Fig. 1B).

### 3.2. Development of machine learning-based prognostic models

**Figure 2.**
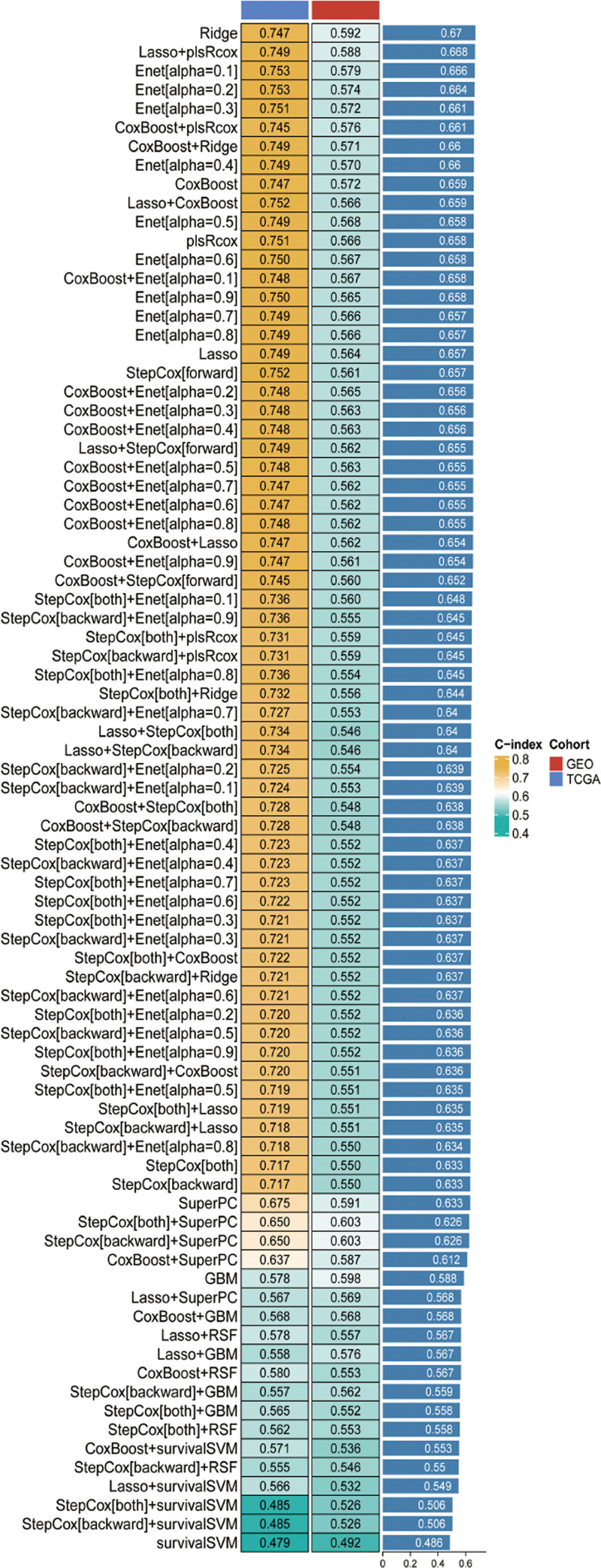
Construction and evaluation of the CRC prognostic model. CRC = colorectal cancer.

Among the various models tested, the Ridge regression model demonstrated the most robust performance, with a mean C-index of 0.67 (Fig. 2). This high C-index underscores the model’s predictive accuracy in survival analysis. Participants were stratified into high and low IRGRS groups based on the median expression levels of these genes.

### 3.3. Prognostic utility of the IRGRS score in patient survival outcomes

**Figure 3.**
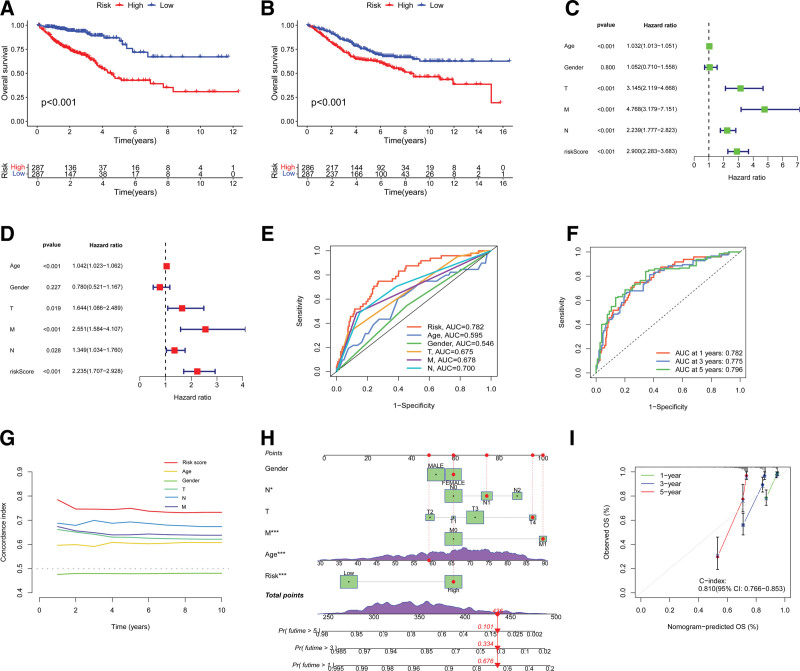
(A) The Kaplan–Meier curve for overall survival (TCGA). (B) The Kaplan–Meier curve for overall survival (GO). (C) Univariate Cox regression analysis. (D) Multivariate Cox regression analysis. (E) ROC curve analysis with AUC for prognostic factors. (F) Time-dependent ROC curves and AUC comparison. (G) The impact of various factors on the concordance index over time. (H) A nomogram integrating the IRGRS with clinical variables, providing a quantitative method of predicting the probability of 1-, 2-, and 3-year overall survival. (I) Calibration plot. *P < .05, **P < .01, ***P < .001. AUC = area under the curve, GO = Gene Ontology, IRGRS = immune-related gene risk score, ROC = receiver operating characteristic, TCGA = The Cancer Genome Atlas Program.

Patients classified within the high IRGRS score cohort displayed significantly diminished overall survival rates compared to those in the low IRGRS score cohort, as determined by the log-rank test (*P* < .001; Fig. 3A, B). Univariate Cox regression analysis indicated that an elevated IRGRS score is significantly associated with an increased risk of mortality (hazard ratio > 1, *P* < .001; Fig. 3C). This correlation persisted in multivariate Cox regression, establishing the IRGRS score as an independent prognostic marker of survival after adjustment for various clinical parameters (Fig. 3D). To augment clinical decision-making, we constructed a prognostic nomogram that integrates clinicopathological variables to forecast individual survival probabilities at the 1-, 2-, and 3-year marks (Fig. 3H). The calibration plot confirmed the nomogram’s precision in aligning predicted survival probabilities with observed outcomes (Fig. 3I). Collectively, these findings underscore the IRGRS score as a potent predictor of survival, refining prognostic models and informing clinical practice.

### 3.4. IRGRS score and immune cell infiltration and TME

**Figure 4.**
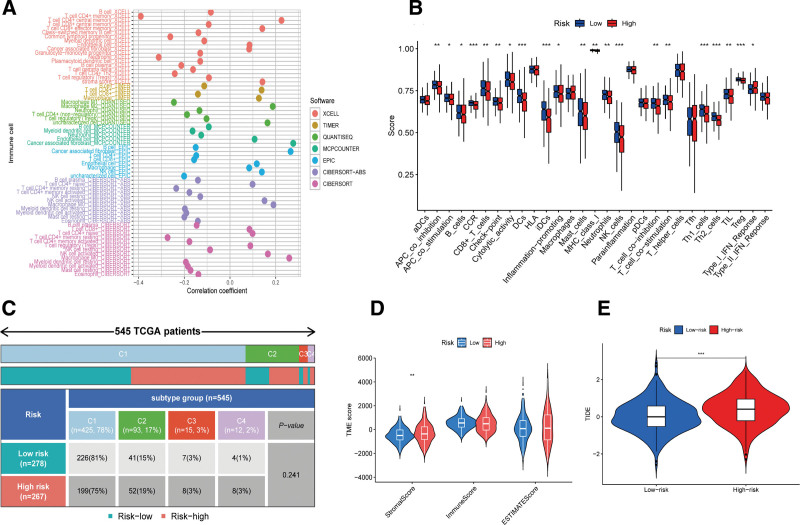
(A) Correlations between immune cell infiltration levels and the IRGRS score. (B) Immune cell infiltration. (C) The distribution of individuals with high and low IRGRS scores across various immune subtypes. (D) TME score. (E) TIDE score. IRGRS = immune-related gene risk score, TIDE = tumor immune dysfunction and exclusion, TME = tumor microenvironment.

Employing tools such as CIBERSORT-ABS, CIBERSORT, EPIC, MCPCOUNTER, QUANTISEQ, XCELL, and TIMER, we established correlations between the IRGRS score and the infiltration levels of a spectrum of immune cells, with varying degrees of positive and negative associations (Fig. 4A). The single-sample gene set enrichment analysis delineated differences in immune-related functions, revealing significant reductions in antigen-presenting cell co-inhibition, co-stimulation, cytolytic activity, inflammation-promoting, T-cell co-inhibition, T-cell co-stimulation, and type-I-interferon-response in the high IRGRS score group compared to the low rating group (Fig. 4B). In addition, the infiltration levels of B cells, CCR, CD8+ T cells, DCs, iDCs, Mast cells, neutrophils, NK cells, Th1 cells, Th2 cells, and TIL were diminished in patients with elevated IRGRS scores (Fig. 4B). However, no significant differences in immune subtype distribution were observed between the high and low IRGRS score groups (Fig. 4C).

TME analysis using the ESTIMATE algorithm indicated that patients with high IRGRS scores had increased stromal scores, suggesting a higher presence of stromal cells in their TME (Fig. 4D). This elevated stromal cell score, indicative of greater stromal cell content in tumor tissue, may be associated with tumor progression and metastatic potential. The high IRGRS score group also exhibited a higher TIDE score than the low-score group (Fig. 4E), implying a potential enhanced capacity for immune evasion and, consequently, a poorer response to immune checkpoint inhibitor therapy and a more adverse prognosis.

### 3.5. Gene set enrichment analysis

**Figure 5.**
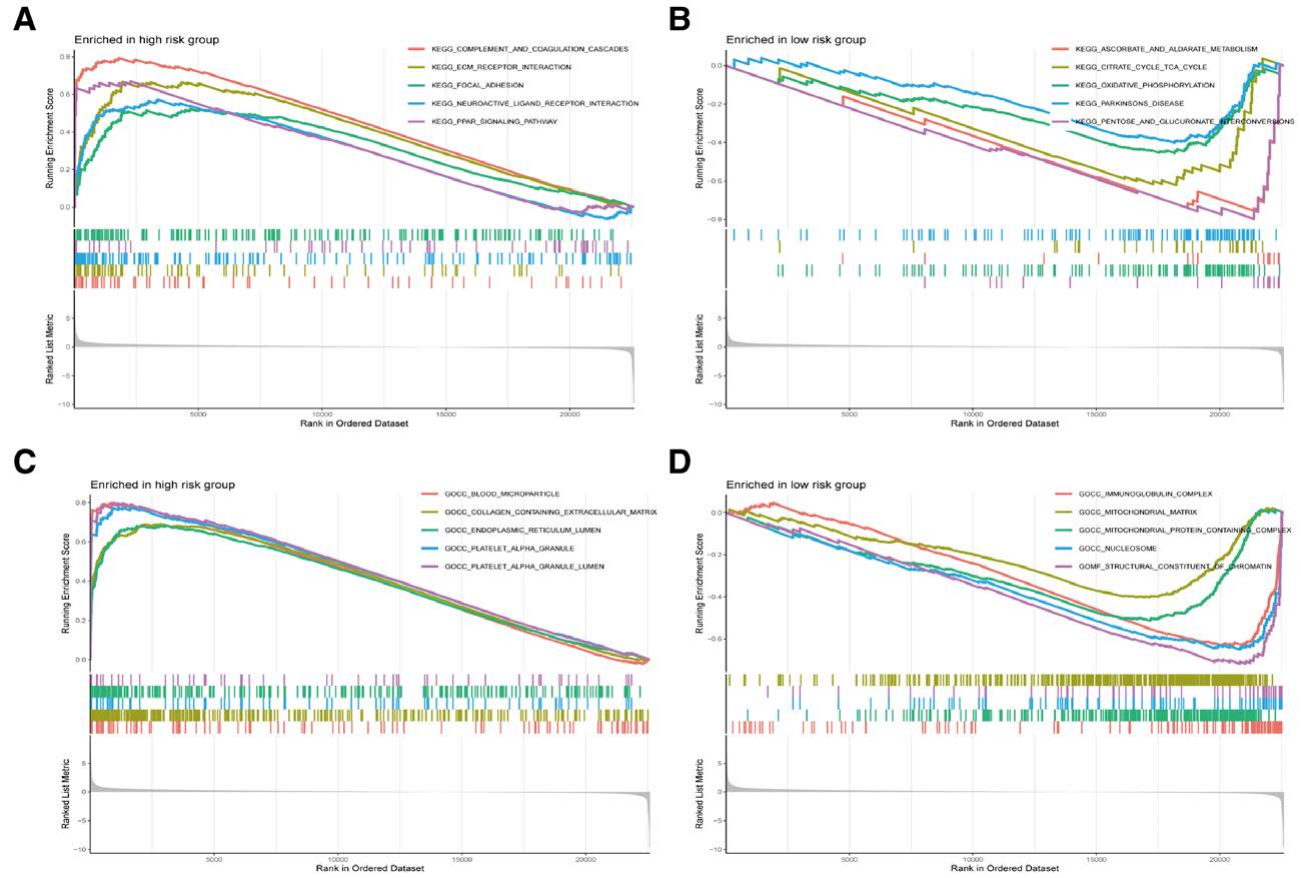
GESA analysis. (A) KEGG pathways enriched in high-risk group. (B) KEGG pathways enriched in low-risk group. (C) GO terms enriched in high-risk group. (D) GO terms enriched in low-risk group. GO = Gene Ontology, GSEA = gene set enrichment analysis, KEGG = Kyoto Encyclopedia of Genes and Genomes.

GSEA was conducted to delineate the most significantly enriched functional terms distinguishing high-risk from low-risk patient cohorts. Our analysis revealed an enrichment of the complement and coagulation cascades in the high-risk group, pathways integral to the immune system’s inflammatory responses, immune regulation, and cell lysis (Fig. 5A). Conversely, pentose and glucuronate interconversions were enriched in the low-risk group (Fig. 5B). Gene Ontology enrichment analysis indicated that the mitochondrial matrix, mitochondrial protein complex, structural components of chromatin, and nucleosome were enriched in the low-risk group (Fig. 5C, D). In contrast, the endoplasmic reticulum lumen, blood microparticles, and lumen of platelet alpha granules were enriched in the high-risk group.

### 3.6. Correlation between IRGRS score and immune checkpoint expression

**Figure 6.**
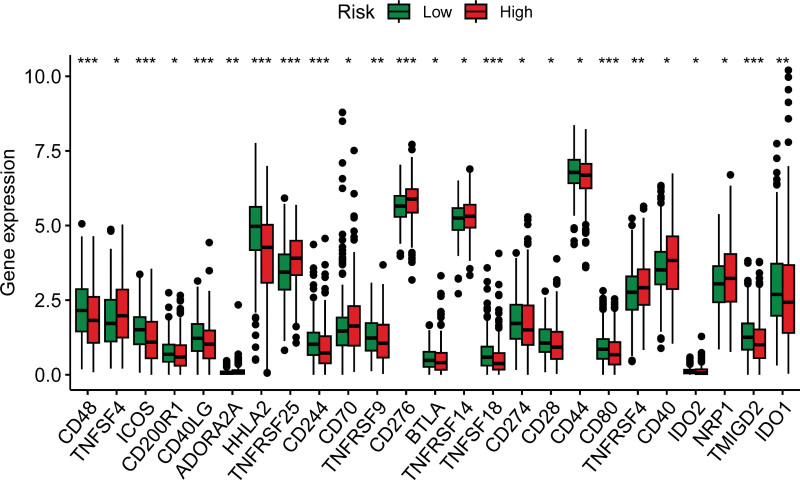
The levels of immune checkpoints.

Subsequently, we investigated the correlation between IRGRS scores and the expression levels of immune checkpoints, as well as their potential impact on drug sensitivity. Patients with high IRGRS scores exhibited significantly increased expression of immune checkpoints such as *CD40*, *NRP1*, *TNFRSF4*, *CD70*, *CD276*, *TNFRSF25*, and *TNFSF4* compared to the low-score group (Fig. 6). Conversely, the expression of immune checkpoints, including *CD48*, *ICOS*, *CD40LG*, *HHLA2*, *CD244*, *TNFSF18*, *CD80*, *TMIGD2*, and *IDO1*, was significantly decreased in patients with high IRGRS scores. This observation suggests a potential link between IRGRS and differential responses to checkpoint immunotherapy, highlighting the IRGRS score’s role in predicting treatment outcome.

### 3.7. Effect of IRGRS score on drug sensitivity

**Figure 7.**
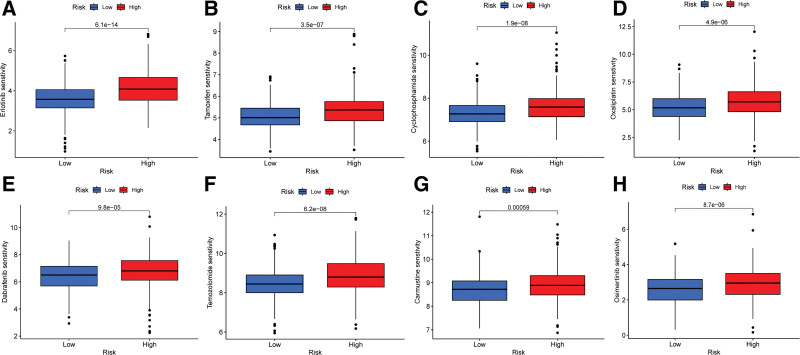
Drug sensitivity of chemotherapeutic agents. (A) Erlotinib, (B) tamoxifen, (C) cyclophosphamide, (D) oxaliplatin, (E) dabrafenib, (F) temozolomide, (G) carmustine, and (H) osimertinib. *P < .05, **P < .01, ***P < .001.

We conducted an analysis to assess the differential sensitivity to a panel of chemotherapeutic agents across patient groups defined by their IRGRS scores, as depicted in Figure 7. Notably, patients assigned to the high IRGRS score group exhibited enhanced sensitivity to a range of chemotherapeutic agents, including carmustine, cyclophosphamide, dabrafenib, erlotinib, osimertinib, oxaliplatin, tamoxifen, and temozolomide. These findings underscore the IRGRS score’s potential as a biomarker for personalized therapeutic strategies, highlighting its utility in predicting drug responsiveness and informing treatment decisions.

## 4. Discussion

With the relentless progress in CRC diagnostics and therapeutics, treatment paradigms have transitioned from one-size-fits-all approaches to personalized, integrated strategies that combine local and systemic therapies tailored to individual patients.^[12]^ Tumor immunotherapy has emerged as a significant therapeutic modality across a spectrum of cancers, leveraging the body’s immune system to recognize, attack, and eradicate cancer cells.^[13]^ The overarching goal of this therapeutic strategy is to augment or mimic the immune system’s innate anticancer capabilities.^[14]^ Immunotherapy encompasses a range of approaches, including immune checkpoint inhibitors, which are drugs designed to block the signaling pathways that cancer cells exploit to evade immune detection, such as PD-1/PD-L1 and CTLA-4 inhibitors.^[15,16]^ However, the efficacy of immunotherapy is variable, and only a subset of patients derives benefit from this treatment.^[17]^ Identifying and selecting biomarkers that can predict the differential efficacy of tumor immunotherapy has become a focal point in research.^[18]^ Effective biomarkers will assist clinicians in devising treatment plans and provide theoretical underpinnings for the variability in the efficacy of tumor immunotherapy.^[19]^ IRGs play a pivotal role in modulating immune responses and predicting responses to immunotherapy.^[20]^ By analyzing these genes, physicians can prognosticate patients’ potential responses to specific immunotherapies. Consequently, IRGs are promising candidates as biomarkers for identifying differences in the efficacy of tumor immunotherapy and predicting patient outcomes.^[21]^ This study employs robust methods to screen immune-related prognostic genes and utilizes them as pertinent biomarkers, offering new targets and insights for the treatment of CRC and the enhancement of patient prognosis.

In this study, we identified a cohort of 48 prognostically associated genes pivotal for model construction. These genes have been implicated in the modulation of immune responses and are hypothesized to be intricately linked to the prognosis of cancer. Notably, *CD1A* and *CD1B*, which are involved in antigen presentation, may modulate tumor immune surveillance.^[22]^ Chemokines such as *CXCL1*, *CXCL2*, *CXCL3*, *CCL28*, *CCL11*, and *CCL24* are suggested to enhance the recruitment of inflammatory cells, thereby influencing the inflammatory milieu of the TME.^[23]^ *IL13RA2* and *IL20RB* are associated with immune regulation.^[24]^ *FABP4* and *CRABP2*, among others, are implicated in the metabolism of fatty acids and vitamin A, potentially impacting the energy metabolism of tumor cells.^[25,26]^ The multifaceted relationship of these genes with CRC prognosis encompasses aspects such as tumor growth, invasion, metastasis, immune response, and therapeutic response.

In our study, patients with elevated IRGRS scores were associated with significantly higher TIDE scores compared to those with lower IRGRS scores. The TIDE score is a prognostic indicator used to forecast the responsiveness of tumors to immune checkpoint inhibitor therapy. An elevated TIDE score may indicate the presence of immunosuppressive cells, such as regulatory T cells (Tregs), or immunosuppressive molecules like PD-L1 within the TME, which can dampen the immune response.^[27]^ It also suggests a heightened potential for immune evasion by the tumor.^[28]^ Furthermore, patients with a high TIDE score may exhibit reduced infiltration of effector T cells, such as CD8+ T cells, into the TME, signifying a compromised immune surveillance function.^[29,30]^ These factors can enable the tumor to resist immune attacks, leading to suboptimal therapeutic outcomes and, consequently, a poorer prognosis for patients. Analysis of immune cell infiltration using the CIBERSORT algorithm confirmed that Tregs infiltrate more in the TME of patients with higher IRGRS scores, while effector T cells, such as CD8+ T cells, infiltrate less. Therefore, CRC patients with high IRGRS scores who also exhibit high TIDE scores are likely to experience adverse outcomes.

In our subsequent analysis, we employed the TME score assessment to delineate the intricacies of the TME. Notably, patients exhibiting elevated IRGRS scores were found to possess a heightened Stromal Score, potentially attributable to the augmented activity of stromal cells, particularly cancer-associated fibroblasts, within the TME.^[31,32]^ These cells are implicated in the facilitation of tumorigenesis, invasion, and metastasis, and may modulate drug delivery, immune cell infiltration, and tumor metabolism.^[33]^ Stromal cells are known to secrete a plethora of cytokines and growth factors, thereby reshaping the TME.^[34]^ We hypothesize that an elevated stromal score may be indicative of an immunosuppressive microenvironment, which could impede the efficacy of immune cells in targeting the tumor, thereby enabling immune evasion by the tumor. Consequently, a high stromal score may correlate with increased resistance to chemotherapy, radiotherapy, and targeted therapies, as stromal cells might shield tumor cells from the cytotoxic effects of these treatments through diverse mechanisms.^[35]^ In addition, stromal cells are implicated in tumor angiogenesis and inflammatory responses,^[36]^ suggesting that a high stromal score reflects heightened activity of these processes in tumorigenesis. Interestingly, no discernible difference in ImmuneScore was observed between patients with high and low IRGRS scores. This observation, coupled with the analysis of immune cell infiltration revealing an increase in Treg cell infiltration, leads us to surmise that tumors may suppress immune responses by secreting immunosuppressive factors or inducing Tregs, resulting in an unchanged immune score. The stromal score emerges as a pivotal biomarker in the formulation of therapeutic strategies; however, its prognostic significance in relation to tumor outcomes necessitates further elucidation through clinical and experimental research.

GSEA has unveiled a significant enrichment of the complement and coagulation cascades within the high-risk cohort. The activation of these cascades is intricately linked to inflammatory responses, immune regulation, and cytolysis, and is posited to amplify immune cell activation and inflammation through protease-activated receptor (PAR) signaling. PAR1, a prototypical PAR, mediates cellular responses to thrombin, a principal effector of the coagulation cascade.^[37,38]^ Furthermore, the activation of these cascades may precipitate shifts in the immune microenvironment, influencing immune cell composition and potentially fueling tumor progression.^[39]^ This cascade activation could be indicative of disease severity and clinical outcomes, exerting an influence on disease progression and therapeutic response by modulating the immune system and the microenvironment.

Patients with elevated IRGRS scores demonstrate a pronounced upregulation in the expression of key immune checkpoints, including *CD40*, *NRP1*, *TNFRSF4*, *CD70*, *CD276*, *TNFRSF25*, and *TNFSF4*. This observation suggests a heightened role for these checkpoints in patients with high IRGRS scores, potentially influencing their responsiveness to immune checkpoint blockade therapy. The differential expression of immune checkpoints in relation to IRGRS scores may modulate patients’ responses to such therapies, underscoring the importance of these molecules in the context of immunotherapy efficacy.^[40]^

In this study, we meticulously screened for genes exhibiting statistically significant differential expression between tumor and normal tissues. We then proceeded to conduct a survival analysis on these differentially expressed genes to identify those associated with prognosis. Through this rigorous selection process, we pinpointed 48 genes that were instrumental in constructing a machine learning model predictive of CRC prognosis. External validation utilizing datasets from the GEO confirmed the model’s efficacy in prognostic prediction and drug sensitivity assessment.

Our approach embraced a multigene combination strategy, integrating recent advancements in immunotherapy. Employing 101 machine learning algorithms, we developed a prognostic model that enhances the precision and reliability of cancer outcome predictions. By harnessing IRGs to delineate TME and immune infiltration characteristics, as well as to forecast treatment responses, we employed a suite of tools and algorithms, including CIBERSORT-ABS, CIBERSORT, and EPIC, to assess immune cell infiltration. This comprehensive approach aids in deepening our understanding of the TME and immune responses.

However, this study is not without limitations; the analysis results have yet to be in vivo validated, and the biological functions require further elucidation. The study’s reliance on bioinformatics and machine learning analyses may not fully encapsulate the complex biological interactions under in vivo conditions. Future work will involve the collection and expansion of clinical samples and attempts to validate the model’s accuracy through external experiments.

Supplemental Digital Content “Supplementary Material” is available for this article.

## Author contributions

**Conceptualization:** Binyan Liu, Huimin Li, Mengdi Hao, Jia Zeng.

**Data curation:** Binyan Liu, Wenjie Li.

**Formal analysis:** Binyan Liu, Wenbin Huang.

**Investigation:** Binyan Liu, Dajin Yuan.

**Methodology:** Binyan Liu, Huimin Li, Xiaoli Liu.

**Project administration:** Binyan Liu, Huimin Li.

**Resources:** Binyan Liu, Yubing Zhu.

**Software:** Binyan Liu, Huimin Li.

**Supervision:** Binyan Liu, Mengdi Hao, Xiaoli Liu.

**Validation:** Binyan Liu, Mengdi Hao.

**Visualization:** Binyan Liu.

**Writing – original draft:** Binyan Liu.

**Funding acquisition:** Lei Ding.

**Writing – review & editing:** Lei Ding.


