# Supplementary material for: Survival prediction of colorectal cancer using 101 machine learning methods based on immune-related genes: A machine learning study
Source: Medicine (Baltimore). 2026 May 22;105(21):e48757. doi: 10.1097/MD.0000000000048757 (PMC13201052; doi:10.1097/MD.0000000000048757)
Supplement: Supplementary file 1 [file medi-105-e48757-s001.doc]

Original Contribution

This study makes three key contributions to colorectal cancer (CRC) prognostication: (1) Methodological innovation: We developed a robust consensus model by systematically evaluating 101 machine learning algorithmic combinations via leave-one-out cross-validation, identifying Ridge regression as the optimal approach for immune-related gene risk scoring (IRGRS), which was externally validated across TCGA and GEO cohorts. (2) Biomarker discovery: We curated and validated a 48-gene immune signature derived from differential expression and survival analyses, establishing IRGRS as an independent prognostic factor with superior predictive accuracy (C-index=0.67). (3) Translational integration: We elucidated the biological underpinnings linking IRGRS to tumor microenvironment remodeling (elevated stromal scores, TIDE scores), immune checkpoint expression profiles, and chemotherapy sensitivity, providing a multidimensional framework for precision immunotherapy and personalized treatment stratification in CRC.
